# Effects of White Bualuang (*Nelumbo nucifera* Gaertn.) Extract on Testicular Histomorphometry and Spermatogenic Parameters in Mancozeb-Exposed Rats

**DOI:** 10.3390/biology15100738

**Published:** 2026-05-07

**Authors:** Jiraporn Laoung-on, Ketsarin Intui, Pimchanok Nuchniyom, Kanokporn Saenphet, Churdsak Jaikang, Nopparuj Outaitaveep, Paiwan Sudwan

**Affiliations:** 1 Research Institute for Health Sciences (RIHES), Chiang Mai University, Chiang Mai 50200, Thailand; nop.outaitaveep@gmail.com; 2 Department of Anatomy, Faculty of Medicine, Chiang Mai University, Chiang Mai 50200, Thailand; ketsarin.intui@gmail.com (K.I.); pimchanok.anatomy1@gmail.com (P.N.); 3 Department of Biology, Faculty of Science, Chiang Mai University, Chiang Mai 50200, Thailand; kanokporn.saenphet@cmu.ac.th; 4 Toxicology Section, Department of Forensic Medicine, Faculty of Medicine, Chiang Mai University, Chiang Mai 50200, Thailand; churdsak.j@cmu.ac.th

**Keywords:** fungicide, testis, spermatogenesis, lotus, toxicity, infertility, oxidative stress

## Abstract

Male infertility is an increasing global health concern associated with declining population growth. Mancozeb (MZ) exposure may induce reproductive toxicity through endocrine disruption and oxidative stress, impairing spermatogenesis. This study evaluated the effects of White Bualuang extract (WBE) on sexual behavior, testicular histomorphometry, and spermatogenic parameters in rats exposed to MZ. Thirty mature male rats were randomly assigned to the following five groups (*n* = 6): Control, MZ 500 mg/kg, MZ + 0.55 mg/kg WBE, MZ + 1.10 mg/kg WBE, and MZ + 2.20 mg/kg WBE, for 30 days. Sexual behaviors, relative testis weight, antioxidant properties, and histomorphometry parameters were determined. MZ-exposed rats had significantly decreased courtship behavior, seminiferous tubule diameter, and a tendency toward decreased spermatogenic cell numbers, along with enlarged interstitial spaces. However, pretreatment with WBE, especially at a dose of 0.55 mg/kg, showed improvements in courtship behavior and several histomorphometry parameters and was associated with increased Sertoli cell efficiency and spermatogenic organization compared with the MZ group. WBE showed potential to reduce lipid peroxidation (LPO) and advanced oxidation protein products (AOPP) in MZ-exposed rats, particularly, the 0.55 mg/kg dose improved courtship behavior and reproductive parameters, supporting further investigation of WBE as an antioxidant and potential functional supplement.

## Simple Summary

Male infertility is an escalating global health concern, and exposure to agricultural chemicals such as mancozeb (MZ) may adversely affect reproductive function through the induction of oxidative stress. This study aims to determine whether White Bualuang extract (WBE), a plant-derived antioxidant, could safeguard the male reproductive system in rats subjected to MZ exposure. Rats exposed to MZ displayed reduced courtship behaviors and testicular structural modifications that could negatively impact sperm production. Conversely, pretreatment with WBE improved sexual behavior and several testicular histological features, particularly at the dose of 0.55 mg/kg. These observations imply that WBE might offer protective effects on reproductive function in the context of chemical-induced harm, warranting further investigation as a natural antioxidant.

## 1. Introduction

Male infertility is one of the factors contributing to declining population growth rates and has become an increasing global health concern [1]. Multiple environmental toxicants, occupational stress, harmful lifestyle choices, and agricultural pesticide exposure have been implicated in the decline of male reproductive health [2,3]. Agricultural chemical exposure can interfere with endocrine regulation, spermatogenesis, and testicular structure, ultimately leading to impaired reproductive function [4,5]. However, the specific functional and structural consequences of such exposures on male reproductive capacity remain incompletely understood.

In Thailand, agriculture is a significant occupational sector, in which farmers are often exposed to agrochemicals during crop production, including the prevalent fungicide MZ for the management of fungal infections in fruits and vegetables [6]. MZ is one of the most commercially available of all ethylene bisdithiocarbamates (EBDCs) and is widely used to control fungal pathogens in agricultural and industrial applications [7,8]. Despite its advantages, MZ has been found to contaminate agricultural products and the environment. Although MZ degrades quickly in the environment, recurrent crop cycle treatment may expose farmers to occupational exposure, and inadequate use of PPE in agricultural settings, especially in developing countries, may raise exposure risk [6]. Surveys of Thai farmers reveal that pesticide usage is widespread, with approximately 31.6% also reporting alcohol consumption [9]. These co-existing exposures may independently or synergistically contribute to oxidative stress, immunological dysfunction, and vulnerability to infection, which are associated with male reproductive problems [10,11]. Numerous experimental studies have reported that MZ exposure may induce reproductive toxicity in male animals [12]. While epidemiological evidence in humans is limited, these findings raise reproductive health concerns for populations with chronic exposure, such as farmers. Nevertheless, evidence linking these effects to functional reproductive outcomes and testicular structural alterations remains limited. MZ can be metabolized to ethylenethiourea, a compound that has been reported to disrupt endocrine function and testicular function and induce oxidative stress [5,13]. Oxidative stress plays a crucial role in fungicide-induced male reproductive dysfunction [14]. The testes are particularly susceptible to oxidative damage due to relatively limited antioxidant defenses; thus, excessive production of reactive oxygen species (ROS) can lead to lipid peroxidation, deoxyribonucleic acid (DNA) damage, and apoptosis of germ cells, ultimately impairing sperm production and quality [15,16]. Moreover, oxidative stress can disrupt the function of Sertoli cells, which are essential for supporting germ cell development and maintaining the integrity of the seminiferous epithelium, which reflects the capacity of Sertoli cells to support spermatogenesis [16,17]. Therefore, antioxidant substances may help to reduce oxidative stress, which may help protect the testicular microenvironment and preserve male reproductive function.

Antioxidant intake is an important strategy for preventing oxidative stress [2]. Recently, there has been an increasing interest in utilizing natural plant-derived chemicals as preventive agents against reproductive toxicity [18]. Many therapeutic plants possess phytochemicals with antioxidant capabilities, including flavonoids and phenolic acids, which can neutralize ROS and mitigate oxidative damage [19,20]. Consequently, plant-derived extracts are being extensively examined as potential protective agents against environmental toxins.

*Nelumbo nucifera* Gaertn. (*N. nucifera*) is a member of Nelumbonaceae and is known as Bualuang in Thai [21]. Many parts of *N. nucifera* have been used in Ayurvedic medicine [22]. Previous studies have reported that *N. nucifera* contains neferine and nuciferine, which demonstrated virucidal and antiviral activity against Severe Acute Respiratory Syndrome Coronavirus 2 (SARS-CoV-2) [23], and inhibit the release of tumor necrosis factor alpha (TNF-α) in macrophages stimulated by bacterial infection, thereby reducing inflammation [24]. Furthermore, prior investigations into phytochemicals have revealed that plant extracts rich in phenolic and flavonoid constituents exhibit considerable antioxidant capabilities, encompassing both free radical scavenging and the suppression of lipid peroxidation [20,25,26]. These antioxidant properties might provide protective effects for tissues vulnerable to oxidative stress, including the male reproductive system [26,27]. Recent findings indicate that white lotus petal extract can protect against MZ-induced reproductive toxicity by improving sperm quality and metabolomic profiles [26]. However, its effects on functional reproductive outcomes and detailed testicular structural changes remain unclear. Despite the increasing evidence of the beneficial effects of plant-derived antioxidants, there is insufficient evidence regarding the ability of White *N. nucifera* to alleviate fungicide-induced reproductive damage.

Considering the reproductive toxicity associated with MZ and the antioxidant capabilities of phytochemicals, it is essential to investigate the effects of natural extracts on testicular morphology and spermatogenesis under conditions of exposure to this toxic agent. WBE contains a rich source of antioxidant phytochemicals and can mitigate MZ-induced reproductive toxicity by reducing oxidative stress and preserving testicular structure and function. Specifically, it is expected that WBE attenuates alterations in oxidative stress biomarkers and improves spermatogenesis and sexual behavior parameters in exposed male rats. Therefore, this study builds upon prior research and aims to evaluate the effects of WBE on sexual behavior, testicular histomorphometry, and spermatogenic parameters in rats exposed to MZ, in order to address these knowledge gaps. By strengthening the biological plausibility of WBE’s antioxidant properties and its role in modulating reproductive parameters, this study contributes to the understanding of strategies to mitigate pesticide-associated reproductive toxicity.

## 2. Materials and Methods

### 2.1. Preparation of White Bualuang Extract

WBE from *N. nucifera* petals was prepared following the established protocols [26]. Briefly, the complete petals were collected from an open field in Uttaradit Province, Thailand, during September 2019. The petals were cleaned, dried, ground, and extracted with hot distilled water. The extract sample (WBE) was dried by lyophilization and stored at −20 °C for later experimentation [26].

The extract underwent analysis of its phytochemical contents, with the phenolics and flavonoids composition being determined through a qualitative Liquid Chromatography-Mass Spectrometry (LC-MS) and Proton Nuclear Magnetic Resonance (^1^H-NMR), as detailed in Table 1 [20,26].

### 2.2. Animals and Experimental Design

Thirty sexually mature male Wistar rats (6–8 weeks old, weighing 220–240 g) were purchased from Nomura Siam International Co., Ltd., Bangkok, Thailand. The study protocol was reviewed and approved by the Animal Ethics Committee, Faculty of Medicine, Chiang Mai University (Approval No. 6/2564). Before the experiment, the animals were acclimatized for at least one week under standard laboratory conditions. They were maintained at a controlled temperature of 25 ± 2 °C with a 12 h light/dark cycle and were provided with a standard pelleted diet and filtered water ad libitum.

After acclimatization, thirty male rats were randomly allocated into five groups (*n* = 6 each). All administrations were performed orally via gavage; Group I (control) received olive oil (1 mL/day). Group II (Mancozeb: MZ) was treated with MZ dissolved in olive oil (500 mg/kg); this dose was selected based on the recommended levels for inducing toxicity in animal models in the previous study [5]. Although higher than real-world human exposure, this dose was used to ensure detectable effects; in occupational settings, repeated low-level exposure may still lead to cumulative internal doses, which can result in adverse health effects over time, such as chronic illness or organ damage. The other groups (Group III-V) were fed WBE at 0.55, 1.10, and 2.20 mg/kg, respectively, followed by administration of MZ (500 mg/kg). All treatments were given once daily in the morning (8.00–10.00 AM) for 30 days. The dose of WBE used in this study was chosen to be similar to that in human consumption, as 1.10 mg/kg corresponds to the daily tea consumption in humans [28]. Body weight was recorded before the initiation of the experiment, at three-day intervals during the experimental period, and at the end of the experiment before euthanasia. Subsequently, all rats were euthanized using an overdose of isoflurane, following which the absence of reflexes was confirmed to indicate a deep state of anesthesia, and then, collected blood samples and euthanized via heart puncture. The right caudal epididymis and testis were excised, trimmed, and weighed prior to the sperm count and histology study. Relative organ weights were calculated as (organ weight/body weight) × 100. All experimental procedures were conducted in accordance with institutional guidelines for the care and use of laboratory animals, with efforts made to minimize animal suffering.

### 2.3. Sexual Behavior Testing

Male sexual behavior was assessed on day 28 of the experimentation via observation in a rectangular cage under dim red-light settings, as this time point is sufficient to detect treatment-related effects on the male reproductive system while minimizing variability associated with repeated testing [27,29]. The males were first allowed to acclimate to the testing environment for 10 min. Subsequently, sexually receptive females in the estrous phase were introduced, and mating behavior was recorded for 30 min. The estrous phase of female rats was confirmed by vagina smear and observed under a light microscope (Olympus CH2, Olympus, Tokyo, Japan). The evaluated parameters included courtship behavior, mount frequency (MF), intromission frequency (IF), copulatory efficiency (CE), and intromission ratio (IR). All behaviors were continuously recorded using a video camera throughout the observation period. To verify ejaculation, vaginal smears were obtained from the female rats, and the presence of sperm was examined.

### 2.4. Antioxidant Assay

The supernatant was prepared from the left testis of each rat by homogenization (Glas-Col, Terre Haute, IN, USA) in phosphate-buffered saline (PBS, pH 7.4). The supernatant was used for the determination of LPO, AOPP, advanced glycation end products (AGEs), and ferric-xylenol orange (FOX1) assay. The methods were performed in accordance with the previous study [20,25].

#### 2.4.1. LPO Analysis

A total of 100 µL of supernatant from homogenized testicular tissue was evaluated using the thiobarbituric acid-reactive species (TBARS) assay. Following the experimental procedure, the supernatant was analyzed using a microplate reader (BioTek Synergy H4 Hybrid Microplate Reader, BioTek Instruments, Winooski, VT, USA) at a wavelength of 532 nm [20,25].

#### 2.4.2. AOPP Analysis

A total of 100 µL of supernatant from homogenized testicular tissue was added to a 96-well plate and measured at 340 nm using a microplate reader [20,25].

#### 2.4.3. AGEs Analysis

A 96-well plate consisted of 100 µL of supernatant in each well, which was analyzed for AGEs using a microplate reader at an excitation wavelength of 360 nm and an emission wavelength of 460 nm [20,25].

#### 2.4.4. FOX1 Analysis

The measurement of total oxidant status was evaluated using peroxide content using the FOX1 reagent [20].

### 2.5. Sperm Counts

The right caudal epididymis of each rat was minced and homogenized in 10 mL of Krebs medium (37 °C). Fresh semen suspension (20 μL) was mixed with 20 μL of trypan blue. Then, 10 μL of the mixture was added to a Neubauer hemocytometer (Boeco, Hamburg, Germany). The sperm count was measured in duplicate and expressed as million/mL.

### 2.6. Histology Study of Testis

The right testis was fixed in a 4% formaldehyde fixative solution, dehydrated through a graded series of ethanol, cleared in xylene, and embedded in paraffin wax. The testis was sectioned by microtome (Microm HM 325, Walldorf, Baden-Württemberg, Germany) at a thickness of 4 μm. The testis sections were placed on glass slides, deparaffinized, rehydrated, and stained with hematoxylin and eosin (H&E), and digital photographs were taken under a light microscope at 400× magnification (Olympus CH2, Japan). Twenty round or nearly round seminiferous tubules in stages VII and VIII per rat were collected for diameter, epithelium height, epithelium area, and luminal area measurement. The number of spermatogenic cells and Sertoli cells was analyzed with the ImageJ process version 1.5. All measurements were conducted in a blinded manner to avoid observer bias.

### 2.7. Statistical Analysis

The results are shown in mean ± standard error of mean (SEM). Normal distribution was tested by the Shapiro–Wilk test, and homogeneity of variances was evaluated using Levene’s test prior to statistical analysis. Courtship behavior, AGEs, AOPP, seminiferous tubule diameter, epithelial height, epithelial area, luminal area, type A spermatogonia, pachytene primary spermatocytes, round spermatids, and total number of spermatogenic cells were analyzed using a one-way ANOVA followed by Duncan’s test. MF, IF, CE, IR, LPO, FOX1, body weight, relative testis weight, relative caudal epididymis weight, sperm counts, Sertoli cell nuclei, spermatogonia efficiency, meiotic index, Sertoli efficiency, and SEI were analyzed by the Kruskal–Wallis test and Mann–Whitney U test. All statistical analyses were conducted using SPSS 22.0, and the results with *p* < 0.05 were considered statistically significant.

### 2.8. AI Disclosure Statement

During the preparation of this manuscript, the authors used QuillBot (version 4.53.1) and ChatGPT (version 5.2) for the purposes of grammar correction and used NotebookLM (version 1.37.7) for the purposes of graphic abstract generation.

## 3. Results

### 3.1. Effect of White Bualuang Extract on Sexual Behaviors

The male rats treated with MZ showed a significant decrease in the courtship behavior within 30 min of observation when compared with the control group (*p* < 0.05, Table 2). However, the male rats administered all doses of WBE prior to MZ treatment had significantly improved the courtship behavior from the MZ group, similar to the control group (*p* < 0.05). There were also no significant differences among all treatment groups in MF, IF, CE, and IR (Table 2).

### 3.2. Effect of White Bualuang Extract on Antioxidant Properties

**Figure 1:**
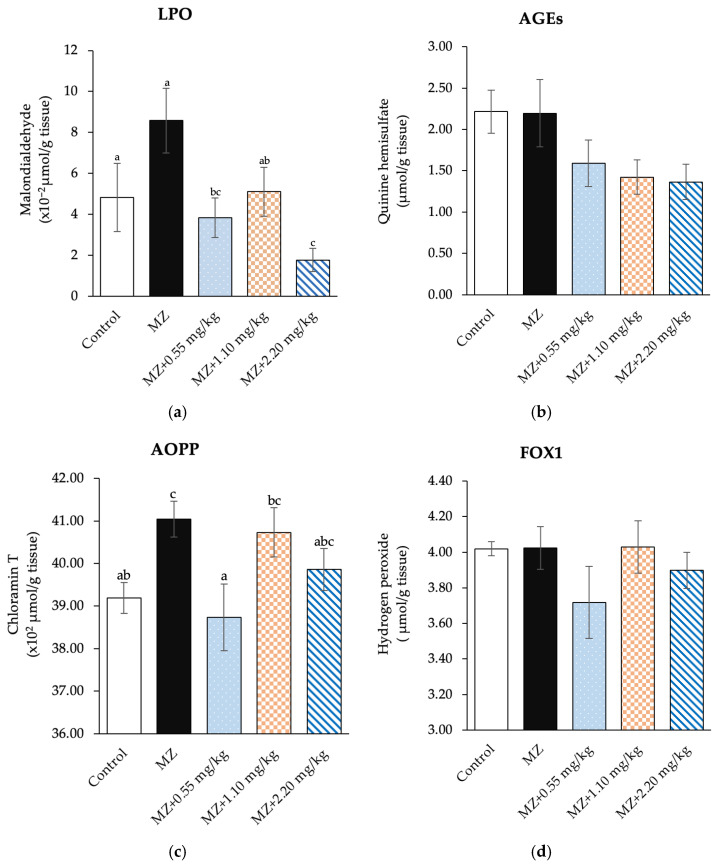
Effect of White Bualuang Extract on levels of selected oxidative biomarkers, LPO (**a**), AGEs (**b**), AOPP (**c**), and FOX1 (**d**) in treated male rats, presented as mean values ± SEM (error bars). ^a,b,c^ Different superscript letters indicate significant differences between groups in column data at *p* < 0.05.

The testicular malondialdehyde levels in male rats treated with MZ tended to increase, but the difference was not statistically significant compared with the control group. In contrast, the male rats administered 0.55 and 2.20 mg/kg of WBE prior to MZ treatment had significantly declined testicular malondialdehyde levels from the MZ-treated group (*p* < 0.05, Figure 1a). Moreover, the MZ-treated group had significantly increased the testicular AOPP levels when compared with the control group (*p* < 0.05). The testicular AOPP levels was significantly decreased in the 0.55 mg/kg of WBE prior to MZ treatment group (*p* < 0.05, Figure 1c). There were also no significant differences in all treatment groups in AGEs and FOX1 (Figure 1b,d).

### 3.3. Effect of White Bualuang Extract on Body Weight, Relative Testis Weight, Relative Caudal Epididymis Weight, and Sperm Counts

The body weight of male rats administered all doses of WBE prior to MZ treatment had significantly decreased when compared with the control group (*p* < 0.05), while the relative testis weight of the MZ-treated group was significantly increased compared with the control group (*p* < 0.05). Male rats administered all doses of WBE prior to MZ treatment showed a significant increase in the relative testis weight when compared with the control and MZ treatment groups (*p* < 0.05). Moreover, sperm counts of the MZ-treated group were significantly decreased when compared with the control group, whereas the sperm counts of all doses of WBE before being treated with MZ had significantly increased in sperm counts when compared with the MZ group and were similar to the control group (*p* < 0.05). However, all groups were not significantly different in relative caudal epididymis weight (Table 3).

### 3.4. Effect of White Bualuang Extract on Seminiferous Tubule Parameters

Histological analysis of seminiferous tubules at stage VII-VIII was used to determine the seminiferous tubule diameter, epithelial height, epithelial area, and luminal area. The seminiferous tubule diameter was significantly decreased in the MZ-treated group when compared with the control group, while the diameter of the seminiferous tubules administered all doses of WBE before being treated with MZ was significantly increased when compared with the MZ group (*p* < 0.05, Table 3). Epithelium height was significantly increased in the 1.10 and 2.20 mg/kg of WBE before being treated with MZ when compared with control and MZ rats (*p* < 0.05). Additionally, the rats receiving WBE at the doses of 0.55 and 2.20 mg/kg before MZ treatment showed a significantly increased luminal area when compared with the MZ group (*p* < 0.05). On the other hand, all groups were not significantly different in epithelium area (Table 4).

### 3.5. Effect of White Bualuang Extract on Testicular Cells and Spermatogenic Indices

The number of type A spermatogonia of male rats treated with MZ tended to decline, but the difference was not statistically significant compared with the control group. In contrast, the male rats administered 0.55 and 1.10 mg/kg of WBE prior to MZ treatment had significantly increased type A spermatogonia when compared with the MZ-treated group (*p* < 0.05). Round spermatids of 2.20 mg/kg of WBE before being treated with MZ were significantly increased when compared with the MZ group (*p* < 0.05). Additionally, the total number of spermatogenic cells tended to decrease in the MZ group, but the difference was not statistically significant compared with the control group. The male rats treated with 0.55 and 2.20 mg/kg of WBE before being treated with MZ had a significantly increased total number of spermatogenic cells when compared with the MZ group (*p* < 0.05). However, all groups were not significantly different in the number of pachytene primary spermatocytes and Sertoli cell nuclei (Table 5).

**Figure 2:**
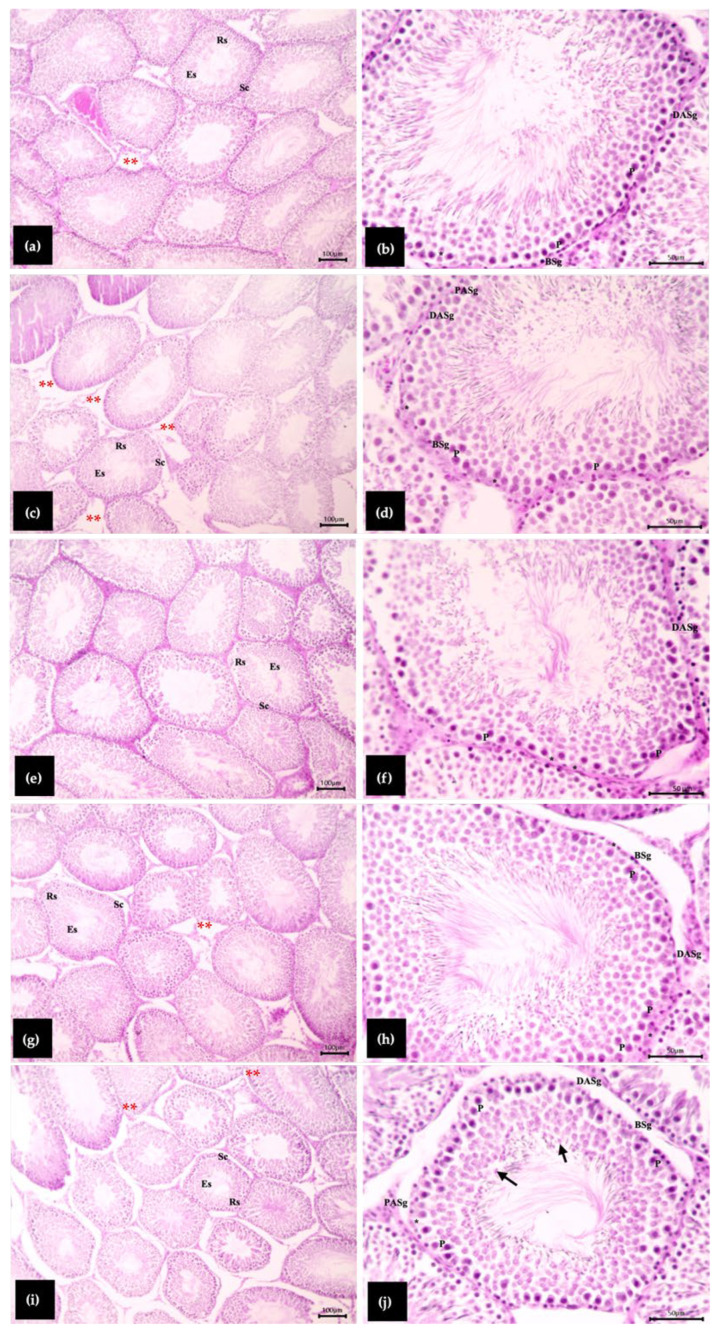
Histological feature of rat testis sections stained with H&E, showing the stages VII or VIII of the seminiferous epithelium. (**a**,**b**) Control group; (**c**,**d**) rats received MZ 500 mg/kg; (**e**,**f**) rats received WNAE 0.55 mg/kg followed by MZ; (**g**,**h**) rats received WNAE 1.10 mg/kg followed by MZ; (**i**,**j**) rats received WNAE 2.20 mg/kg followed by MZ. The black arrow (→): Residual bodies; DASg: Dark type A spermatogonia; PASg: Pale type A spermatogonia; BSg: Type B spermatogonia; Sc: Spermatocytes; P: Pachytene primary spermatocytes; Rs: Round spermatids; Es: Elongate spermatids; *: Sertoli cells; **: Interstitial space.

The spermatogenic indices, including spermatogonia efficiency, meiotic index, Sertoli efficiency, and the Sertoli cell index (SEI), are presented in Table 5. There were no significant differences among all groups in spermatogonia efficiency and meiotic index. In contrast, the male rats treated with 0.55 and 2.20 mg/kg of WBE prior to MZ treatment showed significantly increased Sertoli efficiency and SEI when compared with both the control and MZ-treatment groups (*p* < 0.05, Table 6). The histological appearances of seminiferous epithelium were presented in Figure 2.

## 4. Discussion

MZ is the most commercially available of all EBDCs and is widely used for fungal control in agriculture and industry [30]. It contains heavy metals that can induce male reproductive dysfunction [30]. Natural antioxidants derived from plants have been developed and used to prevent or manage MZ-induced toxicity in the male reproductive system. However, there are limited data regarding the use of plant extracts against MZ toxicity.

Courtship behavior is an important pre-copulatory activity [31] that results from sexual motivation. Sexual motivation is strongly regulated by central dopaminergic pathways and is highly sensitive to oxidative and neuroendocrine disturbances [31]. Moreover, sexual behavior is regulated by a complicated sequence of motor patterns and multisensory impulses [32]. The present study showed that male rats treated with 500 mg/kg MZ for 30 days significantly reduced courtship behavior when compared with the control group during the entire 30 min observation period, reflecting impaired sexual motivation. It is possible that MZ-induced behavioral impairment is mediated by oxidative stress-related neurofunctional disruption, as it is widely recognized that oxidative stress changes dopamine metabolism and neuronal reactivity [32]. However, male rats receiving all doses of WBE pretreatment showed significant improvement in courtship behavior from the MZ group. This result is consistent with the study on *M. oleifera* leaf tea, which was reported to enhance sexual motivation [27]. Polyphenols function as antioxidants and may stimulate the production of dopamine by acting in the hypothalamic region and medial amygdala, thereby facilitating successful sexual behavior in male rats pretreated with WBE, possibly confirming neuroprotective antioxidant function [32,33].

The evaluation of AOPP and the increasing trend of MDA in MZ-induced rats indicated increased protein and lipid peroxidation. Testicular tissue is particularly vulnerable due to its high content of polyunsaturated fatty acids and active mitochondrial metabolism, which is easily prone to oxidative damage [2,34]. ROS disrupt lipid membranes, oxidize proteins, and damage germ cell DNA [14]. Pretreatment of male rats with WBE significantly reduced AOPP and LPO levels compared with the MZ-induced group, particularly at 0.55 mg/kg, suggesting mitigation of testicular damage from oxidative stress. Consistent with previous findings, the white *N. nucifera* extract significantly reduced LPO from the cattle sperm treated with MZ, and its antioxidant properties can be attributed to the properties of white *N. nucifera* extract, which can be explained by the biological activities of phenolic and flavonoid compounds [20]. Phenolics and flavonoids are powerful natural antioxidants that can scavenge free radicals and disrupt LPO chain activities by contributing hydrogen atoms or electrons, and as reducing agents, singlet oxygen quenchers, and metal chelators, they limit biological membrane oxidative propagation [28,35,36]. Therefore, the high total phenolic and flavonoid content of WBE likely underlies its strong antioxidant activity, contributing to the prevention of cellular damage and maintenance of reproductive function under oxidative stress.

The histomorphometric findings of this study provide substantial insights into the alterations to the structure in testicular tissue due to exposure to MZ and also the protective effects of WBE. Although the testis weight in the MZ-treated group was higher than that of the control group, this increase does not indicate improved reproductive function but may reflect pathological changes associated with MZ-induced toxicity. Testicular weight alone is not a dependable measure of spermatogenic activity, as it may be influenced by numerous pathological circumstances, such as interstitial edema, vascular congestion, inflammatory reactions, Leydig cell hypertrophy, or germ cell degeneration [37,38]. Accordingly, an increase in testis weight following toxicant exposure may not mean that spermatogenesis functions more efficiently, but rather that something is wrong with it.

The current study revealed that the elevated relative testis weight in the MZ group coincided with a reduction in seminiferous tubule diameter and a tendency for a decrease in the total number of spermatogenic cells. Histological analyses further supported these findings by showing that the interstitial spaces in the MZ-treated rats were bigger than those in the control group. It is interesting that these areas had a few cells and looked more like fibrous or empty spaces than active interstitial tissue. These changes could mean that the seminiferous tubules become damaged, the seminiferous epithelium begins to collapse, or there is interstitial edema because of oxidative damage caused by toxicants. Previous studies have demonstrated that oxidative stress may damage the blood–testis barrier, impair Sertoli–germ cell junctions, and trigger germ cell apoptosis, ultimately resulting in the degeneration of seminiferous tubules and the expansion of interstitial compartments [16,39]. A decline in seminiferous tubule diameter and epithelium thickness frequently implies reduced spermatogenesis or germ cell deficiency resulting from toxicant exposure [16]. Moreover, rats treated with MZ showed a significant decrease in sperm counts compared with the control group, consistent with previous studies presenting that MZ exposure leads to a significant decline in sperm count and spermatogenic cells in male rats [40], indicating its detrimental effect on spermatogenesis.

An enhancement in seminiferous tubule diameter and epithelial thickness frequently indicates increased germ cell proliferation and effective spermatogenesis [29,41]. In this current study, male rats administered WBE, particularly at doses of 0.55 and 2.20 mg/kg prior to MZ exposure, showed enhancements in sperm counts and seminiferous tubule morphology compared with the MZ group. The improvement in sperm counts in the WBE-treated group compared to the MZ group may be attributed to the bioactive constituents of WBE, which exhibit potent antioxidant properties capable of mitigating oxidative stress and promoting spermatogenesis [26]. The seminiferous tubules demonstrated greater organization, and a tendency towards enhanced spermatogenic cell counts reflected a partial recovery of spermatogenic activity. Moreover, Sertoli efficiency and the Sertoli cell index were significantly enhanced in male rats administered WBE, particularly at doses of 0.55 mg/kg, compared to the MZ-treatment group. Sertoli efficiency and SEI reflect the capacity of Sertoli cells to support and regulate germ cell proliferation, which is related to the spermatogenic activity and efficiency of Sertoli cell supportive functions [17,42], which suggests a beneficial intratesticular microenvironment and enhanced spermatogenic quantity. Moreover, neferine and nuciferine in *N. nucifera*, as alkaloids, presented virucidal and antiviral activity inhibition of SARS-CoV-2 [23] and reduced inflammation caused by the infection [24], which may cause reproductive inflammation. These improvements may be explained by the antioxidant properties of phytochemicals present in WBE, particularly phenolics, flavonoids, and alkaloids, which can scavenge free radicals, reduce lipid peroxidation, and stabilize cellular membranes [20]. In addition, antioxidants may help preserve tight junction proteins and maintain the integrity of the blood–testis barrier, which is essential for supporting spermatogenic cell development [43].

Therefore, WBE demonstrated antioxidant potential that may enhance male reproductive function through improved sperm counts, testicular histology, and spermatogenic organization, indicating that its antioxidant properties could preserve the testicular microenvironment and male reproductive capability.

## 5. Conclusions

In conclusion, WBE demonstrated a protective effect against MZ-induced oxidative stress in male rats, as evidenced by the reduction in LPO and AOPP levels. Additionally, the WBE, especially 0.55 mg/kg, was associated with improvements in courtship behavior and male reproductive functions. These findings suggest that WBE may help mitigate oxidative stress-related reproductive impairment under the conditions of this study. However, elucidating the underlying mechanisms and investigating their potential applicability in other contexts should be further studied.

## Figures and Tables

**Table 1:** Phytochemical components of WBE determined through LC-MS and ^1^H-NMR [20,26].

Determination Methods	Phytochemical Compounds
LC-MS	(+)-delta-Tocopherol
	Kaempferitrin
	Ouabain
	Convallatoxin
	Salasodine
	Isorhamnetin-3-O-rutinoside
	2′, 3, 3′ 4, 4′-pentahydroxy-4′-glucosulchalcone
	4, 8′-Bi ((+)-epicatechin)
	Quercetin-3-O-arabinoglycoside
^1^H-NMR	Myricetin
	Apigenin
	Luteolin
	Ferulic acid
	Caffeic acid
	Ascorbic acid
	Genistein
	Chlorogenic acid
	Naringin
	Ellagic acid

**Table 2:** Sexual behavior parameters of male rats treated with WBE compared with the control and MZ groups.

Group	Sexual Behavior Parameters				
	Courtship (×10^2^ s)	MF	IF	CE (IF/MF)	IR (IF/Total Mount)
Control (*n* = 6)	6.32 ± 0.42 ^a^	12.67 ± 9.48	9.17 ± 6.70	0.45 ± 0.21	0.24 ± 0.11
MZ (*n* = 6)	2.34 ± 0.48 ^b^	16.83 ± 10.51	15.50 ± 10.10	0.15 ± 0.15	0.16 ± 0.10
MZ + 0.55 mg/kg (*n* = 6)	5.82 ± 1.15 ^a^	5.83 ± 3.44	5.17 ± 3.16	0.32 ± 0.20	0.24 ± 0.11
MZ + 1.10 mg/kg (*n* = 6)	6.20 ± 0.84 ^a^	17.67 ± 11.91	17.67 ± 11.91	0.33 ± 0.21	0.17 ± 0.11
MZ + 2.20 mg/kg (*n* = 6)	6.50 ± 1.01 ^a^	1.00 ± 1.00	0.83 ± 0.83	0.14 ± 0.14	0.08 ± 0.08

The data presented mean values ± standard error of mean (SEM). ^a,b^ Different superscript letters indicate significant differences between groups in column data at *p* < 0.05. MF: mount frequency; IF: intromission frequency; CE: copulatory efficiency; IR: intromission ratio.

**Table 3:** Body weight, relative testis weight, relative caudal epididymis weight, and sperm counts of male rats treated with WBE compared with the control and MZ groups.

Group	Body Weight (g)	Relative Testis Weight (g/100 g BW)	Relative Caudal Epididymis Weight (g/100 g BW)	Sperm Counts (×10^6^ Cells/mL)
Control (*n* = 6)	412.50 ± 21.20 ^a^	0.86 ± 0.02 ^a^	0.06 ± 0.01	48.83 ± 3.31 ^a^
MZ (*n* = 6)	379.17 ± 7.12 ^ab^	0.92 ± 0.01 ^b^	0.06 ± 0.01	40.42 ± 1.40 ^b^
MZ + 0.55 mg/kg (*n* = 6)	334.18 ± 21.62 ^b^	1.08 ± 0.07 ^c^	0.07 ± 0.01	49.08 ± 3.12 ^a^
MZ + 1.10 mg/kg (*n* = 6)	357.50 ± 20.20 ^b^	1.00 ± 0.05 ^bc^	0.07 ± 0.01	52.63 ± 4.25 ^a^
MZ + 2.20 mg/kg (*n* = 6)	326.67 ± 10.85 ^b^	1.11 ± 0.04 ^c^	0.06 ± 0.01	49.13 ± 1.42 ^a^

The data presented mean values ± standard error of mean (SEM). ^a,b,c^ Different superscript letters indicate significant differences between groups in column data at *p* < 0.05.

**Table 4:** Seminiferous tubule diameter, epithelium height, epithelium area, and luminal area at stage VII-VIII of male rats treated with WBE compared with the control and MZ groups.

Group	Seminiferous Tubule Diameter (μm)	Epithelial Height (μm)	Epithelial Area (×10^3^ μm^2^)	Luminal Area (×10^3^ μm^2^)
Control (*n* = 6)	350.44 ± 6.94 ^a^	40.59 ± 0.58 ^a^	29.33 ± 1.34	16.89 ± 0.97 ^ab^
MZ (*n* = 6)	309.51 ± 9.56 ^b^	40.70 ± 0.56 ^a^	27.57 ± 1.03	15.16 ± 1.44 ^a^
MZ + 0.55 mg/kg (*n* = 6)	362.19 ± 6.08 ^a^	43.23 ± 0.51 ^b^	33.20 ± 1.24	21.73 ± 1.91 ^b^
MZ + 1.10 mg/kg (*n* = 6)	350.16 ± 6.54 ^a^	42.92 ± 0.73 ^b^	33.35 ± 1.67	19.34 ± 1.09 ^ab^
MZ + 2.20 mg/kg (*n* = 6)	355.20 ± 8.91 ^a^	41.14 ± 0.64 ^a^	32.85 ± 4.15	21.52 ± 2.19 ^b^

The data presented mean values ± standard error of mean (SEM). ^a,b^ Different superscript letters indicate significant differences between groups in column data at *p* < 0.05.

**Table 5:** Numbers of spermatogenic and Sertoli cell nuclei in cross-section of seminiferous tubule at stage VII-VIII of male rats treated with WBE compared with the control and MZ groups.

Group	Type A Spermatogonia (×10^2^ Cells)	Pachytene Primary Spermatocytes (×10^2^ Cells)	Round Spermatids (×10^2^ Cells)	Total Number of Spermatogenic Cells (×10^2^ Cells)	Sertoli Cell Nuclei (×10^2^ Cells)
Control (*n* = 6)	7.35 ± 0.41 ^ab^	6.72 ± 0.58	22.37 ± 0.84 ^ab^	36.44 ± 1.72 ^ab^	1.61 ± 0.20
MZ (*n* = 6)	6.48 ± 0.39 ^a^	5.83 ± 0.25	20.80 ± 1.64 ^a^	33.11 ± 2.06 ^a^	1.40 ± 0.20
MZ + 0.55 mg/kg (*n* = 6)	8.94 ± 0.31 ^c^	7.02 ± 0.40	23.47 ± 1.41 ^ab^	39.43 ± 1.51 ^b^	1.10 ± 0.11
MZ + 1.10 mg/kg (*n* = 6)	7.80 ± 0.11 ^b^	6.73 ± 0.29	20.33 ± 0.88 ^a^	34.86 ± 1.08 ^a^	1.02 ± 0.03
MZ + 2.20 mg/kg (*n* = 6)	7.35 ± 0.39 ^ab^	7.69 ± 0.76	25.38 ± 0.44 ^b^	40.42 ± 0.67 ^b^	1.28 ± 0.17

The data presented mean values ± standard error of mean (SEM). ^a,b,c^ Different superscript letters indicate significant differences between groups in column data at *p* < 0.05.

**Table 6:** The spermatogonia efficiency, meiotic index, Sertoli efficiency, and SEI of male rats treated with WBE compared with the control and MZ groups.

Group	Spermatogonia Efficiency	Meiotic Index	Sertoli Efficiency	SEI
Control (*n* = 6)	0.91 ± 0.06	3.43 ± 0.25	15.08 ± 1.81 ^a^	21.68 ± 3.13 ^a^
MZ (*n* = 6)	0.91 ± 0.05	3.58 ± 0.28	15.54 ± 1.14 ^a^	25.15 ± 2.33 ^ab^
MZ + 0.55 mg/kg (*n* = 6)	0.79 ± 0.06	3.42 ± 0.32	21.92 ± 0.96 ^b^	37.23 ± 2.43 ^c^
MZ + 1.10 mg/kg (*n* = 6)	0.86 ± 0.04	3.04 ± 0.14	20.18 ± 1.31 ^ab^	34.61 ± 1.97 ^abc^
MZ + 2.20 mg/kg (*n* = 6)	1.08 ± 0.15	3.47 ± 0.34	22.11 ± 3.46 ^b^	35.23 ± 5.54 ^bc^

The data presented mean values ± standard error of mean (SEM). ^a,b,c^ Different superscript letters indicate significant differences between groups in column data at *p* < 0.05.

## Data Availability

The authors state that the data supporting the results of this study are available in this article.
